# Surfactant Protein D Recognizes Multiple Fungal Ligands: A Key Step to Initiate and Intensify the Anti-fungal Host Defense

**DOI:** 10.3389/fcimb.2020.00229

**Published:** 2020-05-29

**Authors:** Taruna Madan, Uday Kishore

**Affiliations:** ^1^Department of Innate Immunity, ICMR-National Institute for Research in Reproductive Health, Mumbai, India; ^2^Biosciences, College of Health and Life Sciences, Brunel University London, Uxbridge, United Kingdom

**Keywords:** SP-D, fungi, cell wall, polysaccharides, glycoprotein, mycoses, allergy

## Abstract

With limited therapeutic options and associated severe adverse effects, fungal infections are a serious threat to human health. Innate immune response mediated by pattern recognition proteins is integral to host defense against fungi. A soluble pattern recognition protein, Surfactant protein D (SP-D), plays an important role in immune surveillance to detect and eliminate human pathogens. SP-D exerts its immunomodulatory activity via direct interaction with several receptors on the epithelial cells lining the mucosal tracts, as well as on innate and adaptive immune cells. Being a C-type lectin, SP-D shows calcium- and sugar-dependent interactions with several glycosylated ligands present on fungal cell walls. The interactome includes cell wall polysaccharides such as 1,3-β-D-glucan, 1,6-β-D-glucan, Galactosaminogalactan Galactomannan, Glucuronoxylomannan, Mannoprotein 1, and glycosylated proteins such as gp45, gp55, major surface glycoprotein complex (gpA). Recently, binding of a recombinant fragment of human SP-D to melanin on the dormant conidia of *Aspergillus fumigatus* was demonstrated that was not inhibited by sugars, suggesting a likely protein-protein interaction. Interactions of the ligands on the fungal spores with the oligomeric forms of full-length SP-D resulted in formation of spore-aggregates, increased uptake by phagocytes and rapid clearance besides a direct fungicidal effect against *C. albicans*. Exogenous administration of SP-D showed significant therapeutic potential in murine models of allergic and invasive mycoses. Altered susceptibility of SP-D gene-deficient mice to various fungal infections emphasized relevance of SP-D as an important sentinel of anti-fungal immunity. Levels of SP-D in the serum or lung lavage were significantly altered in the murine models and patients of fungal infections and allergies. Here, we review the cell wall ligands of clinically relevant fungal pathogens and allergens that are recognized by SP-D and their impact on the host defense. Elucidation of the molecular interactions between innate immune humoral such as SP-D and fungal pathogens would facilitate the development of novel therapeutic interventions.

## Introduction

Fungal infections range from asymptomatic, mild mucocutaneous infections to potentially life-threatening systemic infections. With more than a billion people with mild infections, several millions mucosal candidiasis cases and 150 million people with serious fungal diseases, fungi have a huge impact on public health (Bongomin et al., 2017). Cells of the innate immune system (e.g., dendritic cells and macrophages) bind components of fungal cell walls using surface or soluble pattern recognition receptors (PRRs). The C-type lectin receptors and the Toll-like receptors are particularly involved in anti-fungal immunity (Salazar and Brown, 2018). Surfactant protein D (SP-D), a soluble C-type lectin, recognizes several fungal ligands and inhibits their interactions with target host cells (Pandit et al., 2012; Carreto-Binaghi et al., 2016). Importantly, SP-D can activate or inhibit the pro-inflammatory signaling in the target cells via a range of activating or inhibitory receptors (Kishore et al., 2006).

### Surfactant Protein D, a Soluble Collectin

The human SP-D gene is located proximal to the centromere of chromosome 10 in humans and Chromosome 14 in mouse. The primary structure of the SP-D protein comprises of four distinct regions: an N-terminal non-collagenous domain, a triple-helical collagenous region, an α-helical coiled-coil neck region, and the C-terminal carbohydrate recognition domain (CRD) (Kishore et al., 2005). This primary structural organization exists as a trimeric subunit, which can form further oligomeric units up to dodecamers. It is quite usual to visualize, under electron microscopy, fuzzy balls of SP-D proteins in lung washings.

SP-D is produced by different cell types, including type II pneumocytes, non-ciliated bronchiolar cells, submucosal gland and epithelial cells of trachea in the lung. Other sources include ductal epithelial cells in the lacrimal apparatus, mucosal and glandular/ductal epithelial cells in the gastrointestinal tract, skin, male and female genitourinary tracts (Kishore et al., 2005). Vascular endothelial cells in the heart and brain tissues also synthesize significant levels of SP-D (Sorensen et al., 2006; Schob et al., 2013). SP-D expression is considerably increased in a human corneal cell line challenged with *A. fumigatus* via TLR4 signaling, and corneal tissue of rats challenged with *Fusarium solani* (Che et al., 2012a,b; Wu et al., 2015). Such a widespread existence of SP-D in various tissues and fluids and its increased expression in response to fungal pathogens emphasizes its importance as an innate immune surveillance molecule at the mucosal barriers.

**Figure 1:**
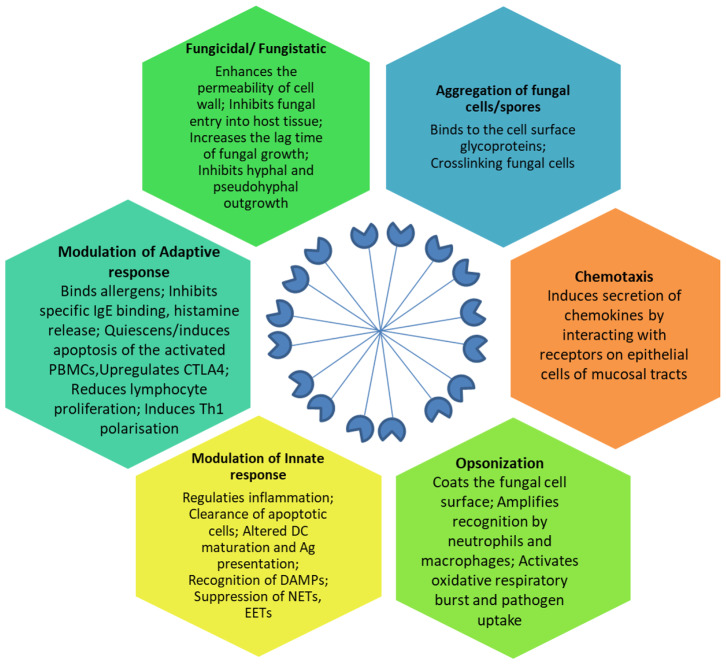
Effector anti-fungal mechanisms mediated by SP-D. The cartoon in the center of the figure depicts the fuzzy ball structure of SP-D formed from several dodecameric structural units. Ag, Antigen; CTLA-4, Cytotoxic T-lymphocyte Associated protein 4; DAMPs, Damage Associated Molecular Patterns; DC, Dendritic cells; EETs, Eosinophil Extracellular Traps; IgE, Immunoglobulin E antibodies; NETs, Neutrophil Extracellular Traps; PBMCs, Peripheral Blood Mononuclear Cells; Th1, T helper 1 lymphocytes.

SP-D mediates immune regulation by direct interaction with multiple receptors on immune and epithelial cells, leading to altered cytokine and free radical production (Jakel et al., 2013; Sorensen, 2018) (Table 1). SP-D stimulates antigen presentation by dendritic cells but inhibits T cell proliferation. SP-D via its collagen domain, binds calreticulin/CD91 receptor complex and activates macrophages. The globular CRD domain promotes an anti-inflammatory effect through a signal inhibitory regulatory protein α (SIRPα) on macrophages (Gardai et al., 2003). Various immunomodulatory mechanisms mediated by SP-D that strengthen host defense against fungi are summarized in Figure 1.

**Table 1:** Various cellular receptors that interact with SP-D.

**Cell type**	**Cellular receptors**	**Function**	**References**
Myeloid cells and soluble form in serum	Cluster of differentiation 14 (CD14)	Inhibition of CD14 interaction with LPS	Sano et al., 2000
Alveolar macrophages, monocyte-derived dendritic cells	CD14/TLR	Inhibition of der p (mite allergen)-induced activation	Liu et al., 2010
Alveolar macrophages	Toll-like receptor 4 (TLR4), MD-2, TLR-2	Inhibition of TLR-2, TLR4 and MD-2 interaction with LPS	Ohya et al., 2006; Nie et al., 2008; Yamazoe et al., 2008
Alveolar macrophages, monocyte-derived macrophages	Signal-regulatory protein-α (SIRP-α)/calreticulin/CD91	SIRP-α interaction with CRD prevents nuclear factor-κB activation, and secretion of inflammatory cytokines Collagen domain interaction with calreticulin/CD91 promotes secretion of inflammatory cytokines	Gardai et al., 2003
Neutrophils	Leukocyte-associated immunoglobulin-like receptor 1 (LAIR-1)	Collagen domain interaction leads to reduction of reactive oxygen species signaling	Olde Nordkamp et al., 2014
Alveolar macrophages	Osteoclast-associated receptor (OSCAR)	Collagen domain interaction results in pro-inflammatory response	Barrow et al., 2015
Eosinophils	Fc receptor γII (FcγRII/CD32)	Inhibitory effect on IgG and serum-triggered eosinophilic cationic protein degranulation by eosinophils	von Bredow et al., 2006
NK cells	NKp46	Secretion of IFN-g and lymph node homing of DCs	Ge et al., 2016
Type II pneumocytes	G Protein-coupled receptor 116 (GPR116)	Regulation of lung surfactant levels	Fukuzawa et al., 2013
Bladder epithelial cells	Uroplakin Ia	Inhibits the adherence and cytotoxicity of uropathogenic *E. coli*	Kurimura et al., 2012
Lung adenocarcinoma epithelial cells	Epidermal growth factor receptor (EGFR)	Suppressing EGF signaling and inhibiting the proliferation and migration	Hasegawa et al., 2015
Dendritic cells and soluble DC-SIGN	DC-SIGN	Reduces HIV-1 capture and transfer to CD4+ T cells	Dodagatta-Marri et al., 2017

Being a calcium-dependent lectin, SP-D binds maltose, glucose, mannose, fucose, galactose, lactose, glucosamine, and N-acetylglucosamine, and to complex carbohydrates on the surface of fungal pathogens (Pandit et al., 2012). The binding of SP-D to a variety of opportunistic fungi results in the direct inhibition of fungal growth, aggregation and enhanced phagocytosis (Pandit et al., 2012). Importantly, the downstream immune response elicited by SP-D also contributes to the reduction/elimination of fungal infection in mice (Pandit et al., 2012).

Here, we review the cell wall ligands of clinically relevant fungal pathogens and allergens that are recognized by SP-D and their impact on the host defense (Table 2). Elucidation of the interactions between SP-D and fungal ligands could lead to the development of novel therapeutic interventions for both allergic and invasive mycoses. We also provide an update on the cellular receptors of SP-D involved in immunomodulation, serum and BAL levels of SP-D, polymorphisms of SP-D, oligomeric forms of SP-D, proteolytic degradation of SP-D and a possible road map to pursue clinical applications of SP-D.

**Table 2:** Ligands of Various Fungi that Interact with SP-D.

**Fungi**	**Fungal ligands recognized by SP-D**	**Source**
*Aspergillus fumigatus*	Glycosylated allergen gp45	3 week culture filtrate
	Glycosylated allergen gp55	3 week culture filtrate
	1,3-β-D-Glucan	Germinating conidia, mycelia
	Melanin (on dormant conidia)	Dormant conidia
	Galactoseaminogalactan (GAG) Galactomannan (GM)	Germinating conidia, mycelia Germinating conidia, mycelia
*Blastomyces dermatitidis*	1,3-β-D-Glucan	Cell wall component of spores
*Cryptococcus neoformans*	Glucuronoxylomannan	Capsule
	Mannoprotein 1	Capsule
*Pneumocystis carinii*	Major surface glycoprotein complex	Cyst and trophozoite
	1,3-β-D-Glucan	Cyst
*Coccidiodes posadasii*	Culture filtrate antigen	Culture filtrate
*Saccharomyces cerevisiae*	Mannoprotein	External cell wall
	1,6-β-D-Glucan	Yeast cell wall

## SP-D Recognizes and Strengthens Host Defense Against *Aspergillus fumigatus*

*Aspergillus fumigatus* is a prominent fungal pathogen in immunocompromised individuals, and can cause invasive aspergillosis. In the immunocompetent host, it causes allergic disorders such as allergic rhinitis, allergic sinusitis, hypersensitivity pneumonitis, and allergic bronchopulmonary Aspergillosis (ABPA).

With a series of *in vitro* and *in vivo* studies, we established that SP-D was relevant in host defense against *A. fumigatus* (Madan et al., 2005a). SP-D bound and agglutinated *A. fumigatus* conidia in a calcium-dependent manner, and enhanced uptake of opsonized conidia by the alveolar macrophages and neutrophils (Madan et al., 1997a). Rat SP-D bound the *A. fumigatus* conidia via CRD; EDTA, mannose, glucose, maltose, and inositol inhibited the binding (Allen et al., 2001). Human SP-D binding to conidia was unaffected by hydrophobic surfactant components. However, SP-D did not increase the association of conidia with rat alveolar macrophages. SP-D-enriched rat BAL fluid inhibited spore binding to extracellular matrix (ECM) proteins and epithelial cells (Yang et al., 2000). Pre-incubation of ECM proteins and epithelial cells with SP-D-enriched BAL fluid prevented the enhancement of spore binding induced by *A. fumigatus* spore diffusate. SP-D localized to *A. fumigatus* surface and stayed bound through the different stages of infection of Calu-3 cells (a human airway epithelial cell line) grown on an air-liquid interface (Ordonez et al., 2019). Importantly, fungal adhesion to the epithelium decreased and fungal clearance by neutrophils increased in the presence of SP-D. Human monocyte-derived macrophages phagocytosed SP-D opsonized dormant conidia more efficiently and upregulated the secretion of pro-inflammatory cytokines (Wong et al., 2018). In a murine model of immunocompromised pulmonary invasive aspergillosis, intranasal SP-D treatment rescued mice from death, concomitant with enhanced local production of protective Th1 cytokines, TNF-α and IFN-γ, and that of protective C-C chemokine, MIP-1α (Singh et al., 2009). Immunosuppressed SP-D gene-deficient mice showed an early death upon conidial challenge, a higher hyphal density and tissue injury in lungs. Treatment with SP-D, or a recombinant fragment of human SP-D composed of trimeric neck and CRD regions (rfhSP-D), reduced the mortality, concomitant with higher IFN-γ to IL-4 ratios in treated SP-D gene-deficient mice (Madan et al., 2010). SP-D gene-deficient immunocompetent mice displayed significantly reduced pro-inflammatory cytokines in the lung upon intranasal challenge with wild-type conidia or melanin ghosts (i.e., hollow melanin spheres) (Wong et al., 2018).

In mice mimicking human ABPA, intranasal treatment with native SP-D (or rfhSP-D) suppressed *A. fumigatus* allergen-specific IgE levels, eosinophilia, pulmonary cellular infiltration and switched the cytokine profile from a pathogenic Th2 to a protective Th1 (Madan et al., 2001). The exogenous SP-D reduced allergen-induced early airway response, bronchial hyper-responsiveness, blood eosinophilia, and Th2 cytokines in murine models of *A. fumigatus* induced allergic asthma possibly by reducing eotaxin levels in the lung (Erpenbeck et al., 2006). SP-D treatment reduced the allergen-induced histamine release from peripheral blood cells. A 9-fold increase in SP-D protein levels with no concomitant changes in SP-D mRNA was observed in the BALB/c mice sensitized intraperitoneally and challenged intranasally with *A. fumigatus* allergenic extract (Haczku et al., 2001). C57BL/6 mice have attenuated *A. fumigatus*-induced allergic airway hyper-responsiveness (AHR) when compared with Balb/c mice owing to a markedly increased SP-D protein expression (Atochina et al., 2003). SP-D gene-deficient mice exhibited intrinsic hypereosinophilia and several-fold increase in levels of IL-5 and IL-13, and lower of IFN-γ to IL-4 ratio in the lungs, suggesting a Th2 bias of immune response (Madan et al., 2005b). SP-D gene-deficient mice were more susceptible than wild-type mice to pulmonary hypersensitivity induced by *A. fumigatus* allergens. Intranasal treatment with SP-D or rfhSP-D was effective in rescuing the *A. fumigatus*-sensitized SP-D gene-deficient mice (Madan et al., 2005b).

### SP-D Interactome on *Aspergillus fumigatus*: Involvement of Both CRD and Collagen Domains

Purified human SP-D bound to the allergens present in the 3-week culture filtrate of *A. fumigatus* as well as purified allergens, gp55 and gp45, in a carbohydrate and calcium-dependent manner but not to the deglycosylated forms of these allergens (Madan et al., 1997b). SP-D inhibited binding of the glycosylated allergens to specific IgE antibodies and induction of histamine release from sensitized basophils. The calcium-activated protein phosphatase, calcineurin, which regulates production of several cell wall molecules, such as 1, 3-β-D-glucan, seems to be a critical factor for SP-D binding to *A. fumigatus* hyphae (Geunes-Boyer et al., 2010). Calcineurin-deficient hyphae [generated via either deletion of the catalytic subunit calcineurin A (Delta cna A) or pharmacologic inhibition by FK506] were not recognized by SP-D. In presence of Caspofungin (which inhibits 1,3-β-D-glucan synthesis) and nikkomycin Z (which inhibits chitin synthesis), SP-D binding to the wild-type strain was enhanced. These observations suggested presence of multiple SP-D ligands, leaving little room for *Aspergillus* to escape immune surveillance mediated by SP-D.

To unravel the SP-D ligands present on dormant conidia, the first pathogen entity that enters the host, various conidial cell wall components were purified and examined for their interaction with SP-D via ELISA (Wong et al., 2018). SP-D significantly bound to melanin pigment (polymer of 1,8-dihydroxynaphthalene), a virulent factor that contributes to immune suppression of the host. Germinating conidia, which lacks in melanin layer, bound to SP-D via two cell-wall polysaccharides, galactomannan (GM) and galactosaminogalactan (GAG). As per the localization of melanin, GM and GAG on the conidial surface, SP-D binding was punctate on the dormant conidia, while it was uniform on germinating conidia. SP-D interacted with melanin via its collagen domain whereas its CRD region was involved in binding to GM and GAG.

## SP-D Binds 1, 3 β-Glucan on *Blastomyces dermatitidis* and Inhibits TNF-α Production

Inhaled spores of *Blastomyces dermatitidis* may cause flu-like symptoms, leading to blastomycosis, an invasive and often serious fungal infection in immunocompromised patients. Bronchoalveolar lavage fluid (BALF)-treated *B. dermatitidis* showed presence of bound-SP-D that was significantly reduced in the presence of 1,3-β-Glucan, a cell wall component of *B. dermatitidis* spores. BALF as well as purified SP-D-supplemented SP-D^−/−^ BALF reduced the TNF-α level from β-Glucan-stimulated murine alveolar macrophages. BALF, pre-incubated with *B. dermatitidis* or β-Glucan, lost the ability to inhibit TNF-α production (Lekkala et al., 2006).

## SP-D Directly Inhibits the Growth of *Candida albicans* and Its Adherence to the Mucosal Epithelial Cells

*Candida albicans* colonizes the mucosal surfaces of humans and is a common member of the human gut and vaginal flora. Immunocompromised individuals develop fatal candidiasis. SP-D bound and agglutinated *C. albicans* (yeast form) in a calcium-dependent manner; this interaction was inhibited by competing sugars such as maltose or mannose. Importantly, incubation with SP-D had a fungicidal effect on *C. albicans*. However, SP-D inhibited phagocytosis of *C. albicans* by alveolar macrophages (van Rozendaal et al., 2000). SP-D strongly bound to *C. albicans* infecting Calu-3 cells grown on an air-liquid interface. Binding with SP-D interfered with adhesion of *Candida* to the epithelium and enhanced neutrophil mediated clearance of opsonized *Candida* (Ordonez et al., 2019). Thus, SP-D seems to facilitate *C. albicans* clearance by making the pathogen readily available for neutrophils away from macrophages and epithelial cells.

## SP-D Binds to Culture Filtrate Antigens of *Coccidioides posadasii*

Coccidioidomycosis is a fungal disease caused by highly virulent, soil-fungus *Coccidioides immitis* or *C. posadasiie*. Inhalation of air-borne arthroconidia leads to initiation of the primary infection. Clinical manifestations include a severe fatal mycosis involving extra-pulmonary tissues. SP-D binds to coccidiodal culture filtrate antigens. Lungs of mice infected intranasally with a lethal dose of *C. posadasii* had significantly reduced levels of SP-D (Awasthi et al., 2004).

## SP-D Recognizes Glucuronoxylomannan and Mannoprotein 1 of *Cryptococcus neoformans* Capsule

*Cryptococcus neoformans* causes infection as a primary human pathogen, or may cause invasive cryptococcosis as an opportunistic pathogen invading the immunocompromised hosts including AIDS patients. Inhalation of acapsular, or sparsely encapsulated cells of *C. neoformans* leads to development of infection with capsule as the chief virulence factor.

SP-D bound and agglutinated acapsular *C. neoformans*, but not the encapsulated form, in a calcium- and sugar-dependent manner (Schelenz et al., 1995). Another study reported that SP-D bound acapsular form with a higher affinity than the encapsulated cryptococci leading to their aggregation (van de Wetering et al., 2004). The cryptococcal capsular components, glucuronoxylomannan (GXM) and mannoprotein 1 (MP1), were identified as SP-D ligands; secreted GXM inhibited SP-D-mediated aggregation of acapsular *C. neoformans*.

SP-D binds and protects *C. neoformans* cells against macrophage-mediated defense mechanisms *in vitro* and *in vivo* (Geunes-Boyer et al., 2009). SP-D binding to *C. neoformans* was calcineurin-independent (Geunes-Boyer et al., 2010). SP-D-deficient mice infected with *C. neoformans* showed reduced eosinophil infiltration, IL-5, fungal burden, and increased survival than wild type control animals (Geunes-Boyer et al., 2012; Holmer et al., 2014). The evidence provided by murine models of fungal infections treated with exogenous native or recombinant human SP-D is direct and reliable. SP-D gene-deficient mice develop increased pulmonary inflammation, emphysema, and surfactant phospholipid accumulations (Botas et al., 1998; Korfhagen et al., 1998; Ikegami et al., 2000, 2005; Wert et al., 2000; Fisher et al., 2002). Further, there is a significant increase in activated lymphocytes, apoptotic alveolar macrophages as well as enlarged, lipid-laden, macrophages that release metalloproteinases and reactive oxygen species (Botas et al., 1998; Korfhagen et al., 1998; Ikegami et al., 2000, 2005; Wert et al., 2000; Fisher et al., 2002). Importantly, oxygen radical release and production of the pro-inflammatory mediators, TNF-α, IL-1, and IL-6, were increased in response to either viral or bacterial pathogens (LeVine et al., 2000, 2001, 2004). These coexisting pulmonary abnormalities of SP-D gene deficient mice complicate the interpretation of challenge models. For example, lymphocyte and macrophage activation along with the increased pro-inflammatory mediators might enhance killing and offset any increase of pathogen survival that results more directly from SP-D deficiency.

## SP-D Mediated Direct Killing of *Histoplasma capsulatum*

*Histoplasma capsulatum* is an intracellular fungal pathogen that causes a self-limiting flu-like illness, and sometimes, a more serious pneumonitis or a chronic cavitary pulmonary infection. In immunosuppressed patients, the fungus can act as an opportunistic pathogen causing a progressive, disseminated disease. Though the ligand on the fungus is not known, exposure to SP-D resulted in increased *H. capsulatum* permeability in a dose-dependent manner, and hence, decrease in viability. However, SP-D did not aggregate *H. capsulatum*, or inhibit the phagocytosis of *H. capsulatum* and growth of macrophage-internalized *H. capsulatum* (McCormack et al., 2003).

## SP-D Binds and Aggregates *Pneumocystis carinii* via Major Surface Glycoprotein Complex and β-glucans

A 3-fold increase in the total alveolar SP-D protein content was observed in the pulmonary fluid samples from *Pneumocystis*-infected humans and rats (Aliouat et al., 1998; Qu et al., 2001). SP-D bound to *P. carinii* via glucose-, mannose-, and N-acetyl-glucosamine-rich major surface glycoprotein complex (gpA), and augmented the adherence of the organisms to alveolar macrophages (O'Riordan et al., 1995). The binding was calcium-dependent and competitively inhibited by maltose > glucose > mannose > N-acetyl-glucosamine, with a higher affinity toward oligomeric forms of SP-D (Vuk-Pavlovic et al., 2001). Maximal SP-D binding was at pH 7.4, with significant inhibition at pH 4. Though SP-D induced aggregation, the phagocytosis of *P. carinii* was not enhanced. SP-D also bound to fungal β-glucans present on Pneumocystis' cystic forms, which are potent stimulators of TNF-α release (Vuk-Pavlovic et al., 1998).

Vuk-Pavlovic et al. generated human SP-D overexpressing transgenic mice that produced approximately 30-50-fold higher level of SP-D and noted that the mice did not had any significant differences in lung morphology and function (Vuk-Pavlovic et al., 2006). These mice were then depleted of CD4^+^ lymphocytes, before and during the *Pneumocystis murina* challenge. SP-D-overexpressing mice exhibited significantly higher fungal burden, with increased levels of TNF-α and macrophage inflammatory protein-2 as well as increased lymphocyte and eosinophil infiltration in BAL on 10 and 14th week (Allen et al., 2001). Though the study inferred increased levels of SP-D led to formation of higher aggregates of *Pneumocystis*, basal levels of other pattern recognition proteins or immunoregulatory molecules were not examined in the SP-D overexpressing mice.

## SP-D Binds Mannoprotein and 1,6-β-D-glucan, Two Cell Wall Components OF *Saccharomyces cerevisiae*

*S. cerevisiae*, an opportunistic pathogen, causes severe infections such as fungemia, endocarditis, pneumonia, peritonitis, urinary tract infections, skin infections, and esophagitis in patients with chronic disease, cancer, and immunosuppression. SP-D bound and aggregated *Saccharomyces cerevisiae* cells and isolated cell walls. Cell wall mannoprotein and 1,6-β-D-glucan of yeast were SP-D ligands but SP-D failed to aggregate chitin. Pustulan [a 1,6-β-linked glucose homopolymer] inhibited SP-D binding to yeast as well as to *A. fumigatus*. F4/80^+^ cells, isolated from the BAL of SP-D gene-deficient mice, internalized considerably less zymosan particles (a glucan with repeating glucose units connected by β-1,3-glycosidic linkages) than F4/80^+^ WT cells (Allen et al., 2001).

## SP-D Reversed the Pulmonary Neutrophilic Infiltration and TNF-α Levels Induced in 1,3-β-Glucan-Modulated Allergic Inflammation

As mentioned above, 1,3-β-glucan is an important fungal ligand recognized by SP-D. Sensitization with 1,3-β-Glucan induced pulmonary neutrophilic infiltration and increased TNF-α level in the BAL of SP-D gene-deficient mice (Fakih et al., 2015). This infiltration was significantly reversed by treatment with rfhSP-D. Thus, a high-dose of SP-D could potentially offer protection against mold-induced exacerbations of allergic asthma.

## Competitive Interaction of SP-D With Other PRRs for the Common Fungal Ligands

Other PRRs such as Dectins, Surfactant Protein-A (SP-A), Mannan-Binding Lectin (MBL), Mannose Receptor (MR), Macrophage inducible calcium dependent lectin receptor (Mincle), Macrophage C-type Lectin (MCL), Complement receptor 3 (CR3), Dendritic Cell-Specific Intercellular adhesion molecule-3-Grabbing Non-integrin (DC-SIGN), Langerin, and melanin-sensing C-type lectin receptor (MelLec) bind to common fungal ligands as SP-D (Goyal et al., 2018). Another complexity in the anti-fungal host defense is brought in by the fact that SP-D binds to other PRRs such as SP-A (Kuroki et al., 1991), TLR-2, TLR-4 (Ohya et al., 2006) and DC-SIGN (Dodagatta-Marri et al., 2017). These complex interactions need further investigation in pre-clinical studies to realize the true translation potential of PRRs.

## Impact of SP-D Genetic Polymorphisms and Proportion of Oligomeric Forms

Genetic polymorphisms of SP-D have been shown to be associated with an increased susceptibility to infections. Allelic variations can influence the quantity and multimerisation of SP-D produced in the serum with differential effects on its binding properties. Individuals with the Thr/Thr (Che et al., 2012b)-encoding genotype had significantly lower SP-D serum levels with predominantly the monomeric form of SP-D than individuals with the Met/Met (Che et al., 2012b) genotype with higher oligomeric forms (Leth-Larsen et al., 2005). However, there has been no study correlating SP-D polymorphisms with susceptibility to fungal infections.

## SP-D Levels in Fungal Allergy and Infections

Serum levels of SP-D are altered in asthma, lung hypersensitivity and pulmonary infection caused by *P. carinii* during AIDS. Median SP-D in BAL was significantly decreased in the lung bacterial infection (12.17 ng/ml) compared with the control group (641 ng/ml), and was below assay limits for the majority of cystic fibrosis children (Postle et al., 1999). In asthmatics and allergics, SP-D levels increased in BAL and serum that went down following corticosteroid therapy (Cheng et al., 2000; Koopmans et al., 2004).

*P. carinii* pneumonia is associated with raised levels of alveolar SP-D, probably as a result of increased expression and accumulation. The synthesis and secretion of SP-D increased with acute injury and epithelial activation (Atochina et al., 2001).

SP-D levels have not been determined for other fungal infections. SP-D serum levels may be a useful and non-invasive diagnostic tool for fungal infections. The successive monitoring of serum levels of SP-A and/or SPD may predict disease activity, although it is presently unclear if these alterations are a cause or consequence of the disease.

## Predisposition of Type-2 Diabetes to Fungal Infections and Serum SP-D Levels

Patients with type-2 diabetes (T2D) exhibited higher serum SP-D concentrations than control subjects (P = 0.006) (López-Cano et al., 2017). There was an inverse association between forced expiratory volume in 1 s (FEV1) and serum SP-D (*r* = −0.265; *P* = 0.029), as well as a significant positive relationship between SP-D concentration and residual volume (*r* = 0.293; *P* = 0.043). Endurance exercise training with improvement in aerobic fitness induced a significant reduction in serum SP-D levels in obese women with T2D (Rezaei et al., 2017). Significantly increased leukocyte SFTPD mRNA levels were observed in hyperglycemic gestational diabetes mellitus (GDM) patients (*P* < 0.05) with a significant positive association with C-reactive protein (Wojcik et al., 2016). Additionally, transcript level of SFTPD also correlated positively with fasting glycemia and insulin resistance. These reports suggest that serum levels of SP-D are increased in T2D patients in response to the increased systemic inflammation and get reduced by endurance exercise. However, none of the studies have evaluated the proportion of various oligomeric forms and functional competence of the serum SP-D for host defense in T2D patients. Importantly, T2D obese patients having respiratory tract infections had lower serum SP-D levels than those who did not have infections (*p* = 0.01) (Jawed et al., 2015). It is likely that in the presence of a respiratory infection, SP-D from the serum moved to the lungs to fight off the pathogen. There are no reports on the association of SP-D levels with respiratory or non-respiratory fungal infections in T2D patients.

## Proteolytic Degradation of SP-D

SP-D levels and proportion of oligomeric forms could be significantly altered by proteolytic degradation. Mildly acidic pH, as might be found in endocytic compartments, may disrupt the lectin-dependent activities of SP-D (Persson et al., 1990). Importantly, SP-D was highly resistant to degradation by a wide variety of neutral proteases *in vitro*, and degradation products have not yet been shown to accumulate under pathological conditions *in vivo* (Brown-Augsburger et al., 1996). Dodecamers of recombinant rat SP-D were not degraded by human leukocyte elastase (HLE) (1 μM) or a variety of secreted mammalian neutral proteases at 37°C in the presence of physiologic calcium concentrations (Crouch, 2000). In the absence of calcium, multimeric SP-D was partially digested by 1,000-fold higher molar concentrations of HLE (50 μM) in a dose- and time-dependent manner with complete digestion happening in 24 h (Griese et al., 2003). Functional studies showed that digested SP-D had lost its calcium-dependent lectin properties, i.e., it neither bound to mannose nor agglutinated bacteria. These studies demonstrate that elastase results in the limited proteolysis of SP-D with loss of its CRD-dependent activities. HLE is present up to 19 U/ml in BAL in patients with cystic fibrosis. Results in patients with CF and high elastase activity in the BAL fluid indicated decreased SP-D in some, but not in all subjects (Griese et al., 2003). In contrast, lung diseases without significant neutrophilic inflammation in BAL fluid are not expected to exhibit degraded SP-D. This observed resistance of native SP-D to proteolytic damage might lead to enhancement of host defense functions on exogenous intranasal administration as validated by the murine model studies (Madan et al., 2001, 2005b, 2010; Singh et al., 2009). Incubation of cell-free BAL fluid with protease IV of *P. aeruginosa* resulted in degradation of SP-D in a time- and dose-dependent fashion; this degradation was inhibited by the trypsin-like serine protease inhibitor N-α-p-tosyl-L-lysine-chloromethyl ketone (TLCK) (Malloy et al., 2005). Degradation by protease IV led to inhibition of SP-D-mediated bacterial aggregation and uptake by macrophages.

## Perspectives

Interaction of SP-D with several fungal pathogens has been established beyond doubt. SP-D binds multiple ligands of several fungi and modulates the immune response of the host geared mostly toward elimination of the pathogen, and alleviation of fungal allergies. Although the studies are limited mostly to respiratory fungal pathogens, with the presence of SP-D in digestive tract mucosa, reproductive tract mucosa, and most importantly skin, it is quite likely that SP-D may impact other fungal infections too.

Functionally competent purified recombinant forms of full length and truncated human SP-D have been evaluated extensively using *in vitro, in vivo*, and *ex vivo* systems, and are available in adequate amounts for therapeutic intervention (Madan et al., 2005a; Mahajan et al., 2013). Intranasal delivery of recombinant SP-D proteins via nebulisers or inhalers would provide the fastest delivery to the site of action, maximal efficacy up to distal airways and half-life (bypassing the proteolytic degradation by other routes of delivery) as evident from the animal model studies (Madan et al., 2001, 2005b, 2010; Singh et al., 2009). rfhSP-D would significantly and immediately reduce the pathogen load as well as restore the immune homeostasis in both allergic and invasive mycoses. In view of the serious adverse effects of available anti-fungal drugs, and a significant therapeutic efficacy and safety of rfhSP-D reported in murine models of allergic and invasive aspergillosis, rfhSP-D as an adjunct therapy may be a more effective option. However, there are no studies yet that have evaluated rfhSP-D in conjunction with standard therapies such as Amphotericin B/ azoles for invasive pulmonary aspergillosis and corticosteroids for allergic pulmonary aspergillosis. Insight into the molecular mechanisms and reproducibility of the preclinical proof-of-concept therapeutic efficacy of rfhSP-D in animal models of fungal infections, strengthen the case for pursuing clinical trials of inhalation formulations of rfhSP-D.

It is important to recognize that there are additional biological functions of SP-D that have been demonstrated already for other diseases. These include: (i) assisting elimination of several viral and bacterial pathogens (Kishore et al., 2005); (ii) enhanced apoptosis of cancer cells (Mahajan et al., 2013) and activated T lymphocytes (Pandit et al., 2016); (iii) attenuation of sepsis-induced pancreatic injury (Liu et al., 2015); (iv) dual role in vascular inflammation and pro-inflammatory disease (Colmorten et al., 2019); and (v) regulation of energy intake and inhibitor of metabolic endotoxemia (Stidsen et al., 2012). This is in addition to its recently discovered involvement in male and female fertility (Kay and Madan, 2016; Rokade et al., 2017). Therefore, it is pertinent to consider that clinical application of SP-D may impact upon more than one facet of health.

## Author Contributions

TM wrote the first draft of the review. UK provided critical suggestions for the manuscript.

## Conflict of Interest

The authors declare that the research was conducted in the absence of any commercial or financial relationships that could be construed as a potential conflict of interest.
